# Factors affecting the delivery of complex rehabilitation interventions in research with neurologically impaired adults: a systematic review

**DOI:** 10.1186/s13643-020-01508-1

**Published:** 2020-11-25

**Authors:** Jain Anne Holmes, Philippa Logan, Richard Morris, Kathryn Radford

**Affiliations:** Division of Rehabilitation, Ageing and Wellbeing, School of Medicine, Univeristy of Nottingham, Nottingham, NG7 2UH UK

**Keywords:** Barriers, Facilitators, Implementation research, Rehabilitation, Long-term neurological conditions

## Abstract

**Background:**

Rehabilitation research does not always improve patient outcomes because of difficulties implementing complex health interventions. Identifying barriers and facilitators to implementation fidelity is critical. Not reporting implementation issues wastes research resources and risks erroneously attributing effectiveness when interventions are not implemented as planned, particularly progressing from single to multicentre trials. The Consolidated Framework for Implementation Research (CFIR) and Conceptual Framework for Implementation Fidelity (CFIF) facilitate identification of barriers and facilitators. This review sought to identify barriers and facilitators (determinants) affecting implementation in trials of complex rehabilitation interventions for adults with long-term neurological conditions (LTNC) and describe implementation issues.

**Methods:**

Implementation, complex health interventions and LTNC search terms were developed. Studies of all designs were eligible. Searches involved 11 databases, trial registries and citations. After screening titles and abstracts, two reviewers independently shortlisted studies. A third resolved discrepancies. One reviewer extracted data in two stages; 1) descriptive study data, 2) units of text describing determinants. Data were synthesised by (1) mapping determinants to CFIF and CFIR and (2) thematic analysis.

**Results:**

Forty-three studies, from 7434 records, reported implementation determinants; 41 reported both barriers and facilitators. Most implied determinants but five used implementation theory to inform recording. More barriers than facilitators were mapped onto CFIF and CFIR constructs. “Patient needs and resources”, “readiness for implementation”, “knowledge and beliefs about the intervention”, “facilitation strategies”, “participant responsiveness” were the most frequently mapped constructs. Constructs relating to the quality of intervention delivery, organisational/contextual aspects and trial-related issues were rarely tapped. Thematic analysis revealed the most frequently reported determinants related to adherence, intervention perceptions and attrition.

**Conclusions:**

This review has described the barriers and facilitators identified in studies implementing complex interventions for people with LTNCs. Early adoption of implementation frameworks by trialists can simplify identification and reporting of factors affecting delivery of new complex rehabilitation interventions. It is vital to learn from previous experiences to prevent unnecessary repetitions of implementation failure at both trial and service provision levels. Reported facilitators can provide strategies for overcoming implementation issues. Reporting gaps may be due to the lack of standardised reporting methods, researcher ignorance and historical reporting requirements.

**Systemic review registration:**

PROSPERO CRD42015020423

**Supplementary Information:**

**Supplementary information** accompanies this paper at 10.1186/s13643-020-01508-1.

## Contributions to the literature

- Research shows developing new rehabilitation for people with long-term neurological conditions is complicated because interventions are complex and are delivered in complex places like hospitals and in the community. Understanding these complexities is important to learn how to overcome them.
- We found a wide range of issues (positive and negative) described in over 40 rehabilitation studies. These start to help us understand the early problems researchers face and how they overcame some of them, which is important planning for future services.
- These findings bring together useful descriptions and contribute to the gaps in the rehabilitation research literature.

## Background

Moving rehabilitation from the research environment into everyday clinical practice requires it to be delivered as intended, in different contexts, achieving the required patient outcomes [1, 2]. Rehabilitation in the United Kingdom (UK) works within complex health and social care systems and involves the delivery of complex interventions [3, 4] to people with long-term neurological conditions (LTNC). LTNCs include conditions such as stroke and traumatic brain injury (TBI). There are between 4.7 and 12.5 million people in the UK living with a neurological condition that negatively impacts their lives [5–7]. Rehabilitation is important because it aims to enable people to reach and maintain optimal physical, sensory, intellectual, psychological and social functioning [8] and is recommended for people with LTNC in the UK [9]. Rehabilitation is measured as part of the National Health Service (NHS) outcomes framework [10] because of its beneficial outcomes for patients and healthcare systems [11].

Rehabilitation interventions cannot change population health outcomes unless adopted [12]. Rehabilitation research is complicated because interventions are complex and this increases the unpredictability of results [13]. Findings do not always translate into improved patient outcomes because of difficulties implementing the intervention in clinical practice [14, 15]. However, there is also a dearth of descriptions of the difficulties faced implementing interventions during trials [16]. This may help to explain why successful single-centre studies do not always progress and scientific discovery halted with interventions that have been determined ineffective when in fact the problem may have been related to its implementation [17]. To improve outcomes for people with LTNC and achieve return on investment in research*,* there is a need to understand factors that affect the implementation of complex interventions [18, 19]. Examining barriers and facilitators associated with delivering interventions in trials is required to learn more about real-world contexts, which may also have a further benefit of reducing waste in research [16, 20].

As an example, vocational rehabilitation (VR) is a form of rehabilitation that supports people with LTNC, to remain in or return to work. Unfortunately, evidence for the effectiveness of VR in people with LTNC is lacking, particularly for TBI [21, 22]. Few studies describe VR for TBI in detail [23] or report its implementation in the context of trials. The exception to this is a UK feasibility RCT where an embedded process evaluation described barriers and facilitators to implementing early VR to people with TBI across three English National Health Service (NHS) sites [24]. The lack of effectiveness and implementation evidence may account, in part, for patchy commissioning of VR services in the UK [25, 26]. Policy makers and commissioners require details about how an intervention will work in different contexts with different populations whilst maintaining optimum outcomes [27, 28]. Trialists can provide assurance about intervention effectiveness by demonstrating that it has been implemented as planned, thus preventing erroneous attribution of effectiveness when interventions are not implemented as planned (type III errors ) [29].

Barriers (hindering delivery) and facilitators (enabling delivery) are often identified together as “determinants” [30]. Factors affecting implementation have been described in over 60 theoretical frameworks [31]. The Conceptual Framework for Implementation Fidelity (CFIF) [32] brings together previous scholarly work understanding how closely interventions are implemented as planned, known as implementation fidelity. In CFIF, fidelity is conceptualised under two domains: adherence and potential moderating factors. Adherence refers to whether an intervention has reached the right recipients (coverage), that recipients then received, and the provider gave the correct intervention content in the right frequency and duration (dose). Moderating factors include the recipient’s response to the intervention, the comprehensiveness of policy description (intervention complexity), facilitation strategies and quality of delivery.

The Consolidated Framework for Implementation Research (CFIR) [33] is considered a meta-theory, bringing together 19 existing theories in a bid to represent every aspect that may be encountered when implementing an intervention. Therefore, CFIR incorporates a wide range of theories in 39 constructs, arranged across five domains (intervention characteristics, outer setting, inner setting, individuals involved and the process of implementation). CFIF and CFIR are used together [34, 35] to explore and describe in detail the complex factors affecting the extent to which an intervention is delivered as intended (fidelity) and those that affect its delivery (implementation).

The literature on the implementation of rehabilitation for adults with LTNCs has not been brought together or described and is not therefore well understood. One exception is a systematic review that focussed on a specific intervention of home-based stroke rehabilitation and investigated determinants (barriers and facilitators) of success [36]. It identified seven studies that provided some information on barriers and facilitators. Siemonsma (2014) found that while none of the studies set out to explicitly identify implementation issues, the use of an implementation framework [37] helped to identify determinants that could then inform suitable implementation strategies in future research.

Differences exist between the implementation of complex interventions within a trial compared *with* clinical practice but little is known about the unique context of the trial setting [16, 38]. For example*,* changing clinicians’ behaviours on unproven interventions is challenging [39], whereas this may be more straightforward with evidence-based interventions. Clinicians often have to deliver interventions in addition to and alongside their usual role without necessarily being experienced in doing this within the research environment [16, 28, 39], whereas those in everyday clinical practice may not have the additional trial-related paperwork or study protocol restrictions. Barriers and facilitators to implementing complex interventions are reported infrequently [39] and even less so in the trial context [16]. Therefore, understanding of what to expect, how to make the most of facilitators and how to overcome barriers is limited. This situation will perpetuate the significant problem of wasting already stretched research funds, that often come from public money, by trialists repeating known but unreported failures in intervention implementation [16, 20]. Understanding implementation issues, will help trialists design and improve strategies to ensure interventions are implemented with fidelity so that the effectiveness of these interventions can be measured with confidence [39, 40].

The aim of this study was to identify the barriers and facilitators affecting the implementation of complex rehabilitation interventions with adults with LTNC within the research context.

## Methods

This review is reported in accordance with PRISMA guidelines [41] and the checklist is available in the supplementary materials. A protocol was developed by the review team (JH, KR, PL) and registered on PROSPERO International Prospective Register of Systematic Reviews [42] (CRD42015020423).

Studies of any design were included if they reported barriers and or facilitators, implementing a rehabilitation intervention, with adults, with LTNCs in developed countries. The WHO’s definition of “rehabilitation” was used: “Rehabilitation is a set of interventions needed when a person is experiencing or is likely to experience limitations in everyday functioning due to ageing or a health condition, including chronic diseases or disorders, injuries or traumas. Examples of limitations in functioning are difficulties in thinking, seeing, hearing, communicating, moving around, having relationships or keeping a job.” [43] Rehabilitation is considered a complex intervention and a complex intervention is characterised by the number and difficulty (e.g. skill requirements) of behaviours required by those delivering the intervention*,* the number of groups or organisational levels targeted by the intervention*,* the number and variability of outcomes*,* the degree of flexibility or tailoring of the intervention permitted [3]. Interventions that were solely related to medication, medical or surgical procedures, or assistive technologies*,* e.g. rehabilitation equipment or e-health, or solely focussed on environmental adaptations, were excluded. No studies were excluded on the basis of research methodology to broaden the scope. Peer-reviewed studies published in English, from database inception until December 2018 were considered, including conference abstracts, and grey literature. Opinion pieces and non-systematic literature reviews were excluded, but they were citation searched. Studies were included from “Developed regions” according to the United Nations’ M49 Standard grouping [44].

Literature searches were developed across a range of databases using medical subject headings and EMTREE thesaurus related to implementation of complex interventions. The search algorithm included the following three main concepts, “barriers and facilitators to implementation”, “long-term neurological conditions” and “complex interventions”. The MEDLINE search algorithm is available in the supplementary materials. The search strategy was adjusted as appropriate for each medical, health, social care and psychology databases from inception to December 2018:

MEDLINE (Ovid: 1946 to current); EMBASE (Ovid: 1980 to current); PsycINFO (Ovid: 1806 to current); CINAHL with Full Text (EBSCOHost: 1981 to current); ASSIA (ProQuest: 1987 to current); AMED (Ovid: 1985 to current); Cochrane Library (Wiley: 1996 to current); Joanna Briggs Institute (Ovid: 1998 to current).

PROSPERO International Prospective Register of Systematic Reviews was searched for ongoing reviews in the same topic area. Research in progress was identified through the UK Clinical Trials Gateway (ukctg.nihr.ac.uk) and the US National Library of Medicine register (clinicaltrials.gov). Citation searches of included studies were undertaken using SCOPUS, Web of Science and Google Scholar. Hand searches of references of relevant papers were conducted. Opportunistic identification of papers were included in the search and marked as not being gathered from other systematised strategies. Searches were recorded in Excel and saved by date on each database where possible. All citations from the database searches were exported to EndNote X8 with duplicates removed and additional results added.

Titles and abstracts were screened by JH against inclusion and exclusion criteria. Full texts were obtained for all titles that met the inclusion criteria or where there was uncertainty. After screening titles and abstracts, two reviewers independently shortlisted studies. A third resolved discrepancies. No additional study information was required from authors. Reasons for excluding studies were documented.

Whilst there are a range of critical appraisal tools for both quantitative and qualitative research appraising the quality of how the primary studies were conducted was not of chief concern for this review. The adjectives describing barriers and facilitators reported in each study were of key importance rather than the primary studies’ effectiveness outcomes. It was expected that a wide range of adjectives would be used that would aid interpretation of reported barriers and facilitators to implementing a complex rehabilitation intervention within a research context, which were not appropriate to assess for quality.

A data extraction table was developed into a Microsoft Excel sheet by the review team. Data was extracted in two stages: (1) descriptive study data and (2) line by line review of units of text (word, sentence, paragraph) describing barriers and facilitators. Extracted data were tabulated onto the Excel sheet to compare data.

A descriptive synthesis was conducted to understand the determinants of implementing interventions in the review in two stages and the review team discussed interpretations to minimise bias. Firstly, units of text were coded by JH on a line by line basis whilst maintaining the correct context of the study. The coding was based on the construct definitions of both frameworks before being individually mapped to constructs of the adapted version of CFIF [45] and CFIR. CFIF was used to identify implementation fidelity and CFIR to identify broader implementation [46]. JH undertook coding and mapping and reported back to the review team to discuss findings and check codes. Differences of opinion about codes and where they were mapped to were discussed before confirming the final map.

Where included studies described data related to study participants, carers, staff delivering the intervention and others, e.g. acceptability, beliefs about the intervention, this was mapped to the relevant constructs on CFIF and CFIR. Where studies did not report data clearly, this was inferred by considering the context of the whole paper and then mapped to relevant constructs. Because studies reported data from different groups using differing methods, it was not feasible to compare them in a meaningful way by for instance counting frequencies.

Secondly, thematic analysis of the units of text was used to reveal more detail about the reported barriers and facilitators and to understand how these were reported. JH conducted the thematic analysis and reported back to the review team to discuss findings and check themes. Differences of opinion about themes were discussed before confirming a final list.

Reviewers were not blinded to publication sources, authors or the countries in which the studies were conducted. However, publisher bias was addressed by maintaining a list of publication sources for all studies to ensure that the search was not limited. Author bias was treated in the same way. Bias towards particular developed countries was addressed by ensuring that the search strategy included a wide range of terms.

## Results

**Fig. 1:**
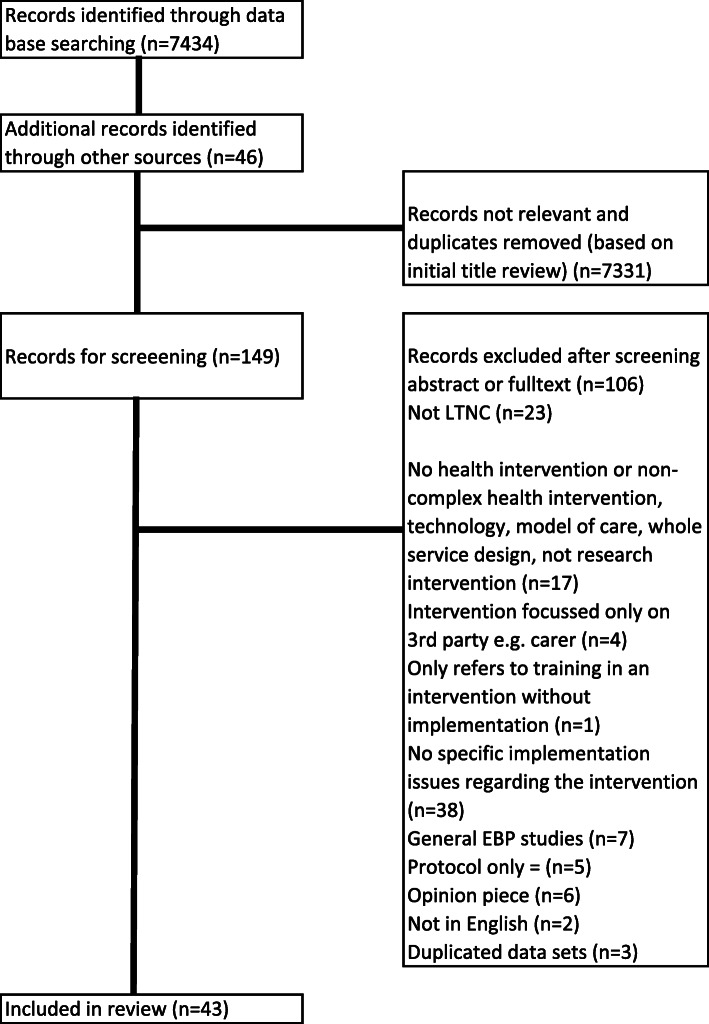
PRISMA flow diagram illustrating selection of studies

The database search returned 7434 records, of which 7331 were excluded. No studies were identified from the grey literature search. Forty-six additional records were identified through author citation search and through recommendations from experts. Full texts were obtained where available for the remaining 149 records and 106 were excluded (see Fig. 1 for reasons). Forty-three studies (including one systematic review) were included in the review.

Details of studies are described in supplementary materials and included: one systematic review (with seven studies that were excluded from the review as primary research); 13 randomised controlled trials (RCTs); eight non-randomised studies (pre- and post-test and mixed methods designs); seven process evaluations (embedded in RCTs); eight qualitative studies (two embedded in a RCT); and five case reports.

The 43 studies were published across 22 different journals between 2006 and 2018. Research was conducted in eight countries:

- Twelve studies from Netherlands [36, 47–57]
- Nine from England [24, 58–65]
- Six from USA [66–71]
- Five from Scotland [38, 39, 72–74]
- Three from Australia [75–77]
- Three from Canada [78–80]
- Two from Germany [81, 82]
- One from Norway [83]
- One international [16]
- One UK study [35]

More than 4000 patients with a range of LTNCs were in receipt of interventions: stroke featured in 22 studies, dementia in seven, Parkinson’s disease in four, multiple sclerosis and mixed LTNCs in three, Huntingdon’s disease in two, motor neurone disease and spinal cord injury in a single study each. The complex interventions delivered were:

- Exercise-based interventions in twelve [16, 50, 51, 58, 67, 68, 70, 71, 73, 76–78]
- Home-based rehabilitation in eight studies [35, 36, 49, 52, 53, 56, 60, 82]
- Psychosocial and educational interventions in seven [48, 54, 57, 64, 74, 75, 83]
- Communication in three [62, 72, 79]
- Continence rehabilitation in three [38, 61, 65]
- Motor imagery interventions in two [47, 59]
- Constraint-induced movement therapy in two [63, 81]
- Vocational rehabilitation [24], music therapy [55], oral care [84], memory aids [66], bathing [69], self-management [80] in a single study each

More than 400 healthcare professionals delivered the interventions and included, in order of prevalence: occupational therapists, physiotherapists, speech and language therapists, psychologists, music therapists, recreational therapists, nurses, physicians, rehabilitation assistants and social workers. Not all studies reported how many professionals, or which profession was involved. Therefore, the numbers are approximate.

Most studies (*n* = 41) reported both barriers and facilitators, while two reported only barriers. Whilst most studies (*n* = 40) described data collection methods, only 10 explicitly examined barriers and facilitators informed by these implementation theories:

- Promoting Action on Research Implementation in Health Services [85]
- Treatment Implementation model [86]
- Framework for the Introduction and Evaluation of Innovations [37]
- Normalisation Process Theory [87]
- Combinations of Interventions [88]
- Theoretical Domains framework [89]
- Consolidated Framework for Implementation Research [33]
- Conceptual Framework for Implementation Fidelity [32]

## Data synthesis

### Stage one—barriers and facilitators mapped to frameworks

**Fig. 2:**
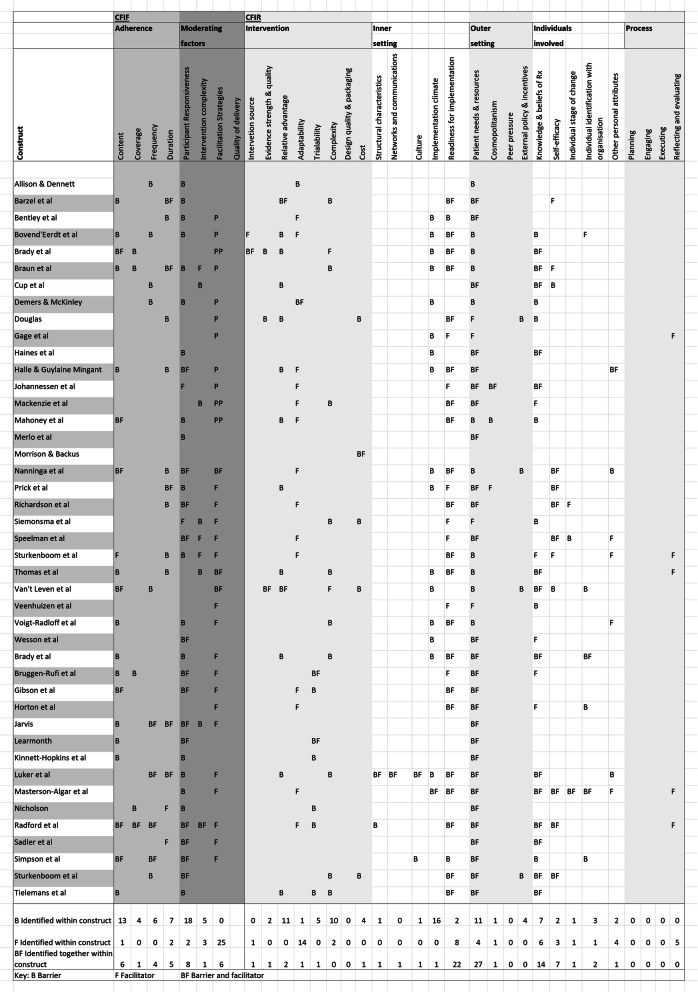
Reported barriers and facilitators mapped to CFIF and CFIR constructs

Figure 2 is a visual representation of reported barriers and facilitators mapped to the constructs of CFIF and CFIR. All studies reported barriers and/or facilitators to implementing the complex intervention under investigation, as this was part of inclusion criteria. Studies reported barriers or facilitators across the implementation frameworks’ constructs and some were co-mapped as both barriers and facilitators.

**Fig. 3:**
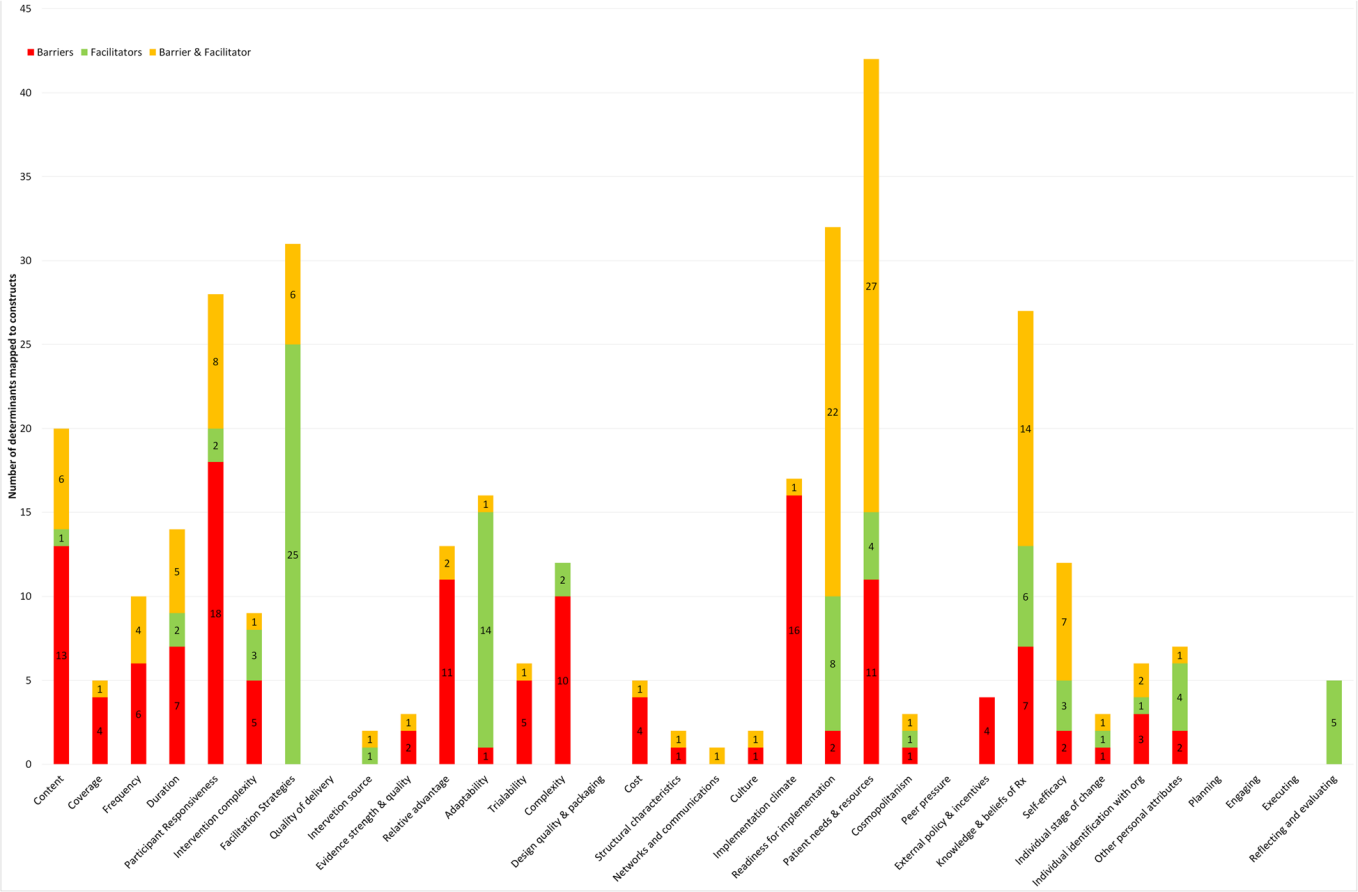
Number of barriers and facilitators mapped to each construct

Figure 3 illustrates the proportion of barriers, facilitators and co-mapped barriers and facilitators in each of the 35 constructs across the two frameworks (Tables 1 and 2). Definitions for the 35 constructs can be seen in the CFIR website resource cfirguide.org.

**Table 1:** List of constructs of CFIF

Conceptual framework for Implementation Fidelity (CFIF)	Domain	Construct
	Adherence	Content
		Coverage
		Frequency
		Duration
	Moderating factors	Participant responsiveness
		Intervention complexity
		Facilitation strategies
		Quality of delivery

**Table 2:** List of constructs of CFIR

	Domain	Construct	Domain	Construct
Consolidated Framework for Implementation Research (CFIR)	Intervention characteristics	*Intervention source*	Outer setting	*Patient needs and resources*
		*Evidence strength and quality*		*Cosmopolitanism*
		*Relative advantage*		*Peer pressure*
		*Adaptability*		*External policy and incentives*
		*Trialability*	Individuals involved	*Knowledge and belief about the intervention*
		*Complexity*		*Self-efficacy*
		*Design quality & packaging*		*Individual stage of change*
		*Cost*		*Individual identification with the organisation*
	Inner setting	*Structural characteristics*		*Other personal attributes*
		*Networks and communications*	Process	*Planning*
		*Culture*		*Engaging*
		*Implementation climate*		*Executing*
		*Readiness for implementation*		*Reflecting and evaluating*

The five constructs with the most determinants mapped to them were “patient needs and resources” (*n* = 42), “facilitation strategies” (*n* = 31), “readiness for implementation” (*n* = 32), “participant responsiveness” (*n* = 28) and “knowledge and beliefs of the intervention” (*n* = 27). Examples of determinants relating to these constructs are described in Table 3.

**Table 3:** Descriptions of determinants most commonly mapped to constructs

Construct	Determinant example
‘Facilitation strategies’	• Experts used by clinicians for support (16, 24, 35, 39, 48, 52, 53, 65). • Specific tools used to deliver an intervention (35, 39, 50, 51, 53, 63, 66, 67, 72, 73, 79) • Naturalistic environments, e.g. home environment, in which to deliver the intervention (49, 69, 72, 79, 80).
‘Patient needs and resources’	• Acceptability, or not, of the intervention by the patient and or carer is part of this construct and was noted in the majority of studies. Reasons for acceptability were not always explored. • Equipment, e.g. a DVD with practice exercises to watch, could not be used by all patients (72); batteries failed (73); unavailability of necessary equipment (16, 35, 38, 55); uncomfortable or inconvenient equipment (63, 68) • Participation difficult due to competing work commitments of patients and or carers (72, 50, 75, 47, 57, 74, 54)
‘Readiness for implementation’	• The organisation’s ability to provide appropriate environments and appointments (16, 48, 62, 63, 64, 69, 74, 79,) or not (16, 35, 54, 55, 62, 64, 74) • Difficulties obtaining staff backfill, or not receiving additional staff for which research funds had been allocated (24, 65) • The availability of appropriate training to deliver the intervention (16, 24, 38, 39, 51, 52, 53, 54, 55, 62, 80, 82) • Not using training resources (24, 62) • Delay between training and starting intervention delivery (24, 54)
‘Participant responsiveness’	• Age, disease severity, physical limitations, sensory impairment, and symptoms such as fatigue were reported across most studies. *Other aspects of participant responsiveness is reported in patient needs and resources above*
‘knowledge and beliefs of the intervention’	• Reports by clinicians that training was only useful if adhered to (39) • Clinicians’ acceptance of the intervention was reported across most studies

The construct “implementation climate” was mapped mostly to barriers (*n* = 16). Examples of these included a failure within the organisation to pre-plan for loss of staff permanently [47], on vacation, other leave, e.g. maternity [47, 59], wards or units under high pressure and unable to dedicate staff time to the intervention delivery and existing ward/department tasks seen as a priority [60, 65, 67, 84], a lack of pre-planning for the impact of clinicians’ needs to travel longer-than-usual distances to visit patients [75, 82], interruption of intervention delivery due to cancelled appointments, being discharged early and hospital admissions [76–79], influential clinician not on-board and advising patients not to engage with the intervention [50], some departments considered the intervention at odds with strategic goals [56], negative attitudes of staff in organisations [16, 24, 38].

Only facilitators were mapped to the construct “reflecting and evaluating” (*n* = 5), which related to time afforded therapists in coaching to gain confidence in intervention delivery and having an allocated person to provide feedback to aid learning [52, 65]. “Facilitation strategies” attracted the most facilitators (*n* = 25) (examples reported in Table 4).

**Table 4:** Themes of barriers and facilitators across studies

	Barriers						Facilitators					
	Non-adherence	Perception of intervention	Attrition	Trial-related	Training	Resources & cost	Adherence	Perception of intervention	Training	Trial-related	Resources & cost	None
Allison & Dennett			✓									✓
Barzel et al	✓		✓				✓					
Bentley et al	✓		✓				✓	✓				
Bovend'Eerdt et al	✓						✓					
Brady et al	✓	✓		✓	✓		✓	✓	✓			
Braun et al	✓	✓	✓	✓			✓	✓				
Cup et al	✓	✓						✓				
Demers & McKinley		✓	✓			✓	✓	✓				
Douglas	✓	✓					✓	✓				
Gage et al		✓		✓			✓	✓				
Haines et al	✓											✓
Halle & Guylaine Mingant	✓						✓	✓				
Johannessen et al		✓		✓			✓	✓		✓		
Mackenzie et al	✓*		✓*				✓	✓				
Mahoney et al	✓			✓			✓	✓				
Merlo et al		✓						✓				
Morrison & Backus						✓					✓	
Nanninga et al	✓	✓					✓	✓				
Prick et al	✓		✓	✓			✓	✓		✓		
Richardson et al	✓		✓				✓	✓	✓			
Siemonsma et al		✓					✓	✓				
Speelman et al	✓	✓					✓	✓				
Sturkenboom et al	✓		✓		✓		✓	✓				
Thomas et al	✓	✓		✓			✓	✓				
Van't Leven et al	✓	✓					✓	✓				
Veenhuizen et al		✓					✓	✓				
Voigt-Radloff et al	✓	✓					✓					
Wesson et al	✓	✓	✓				✓					
Brady et al		✓				✓		✓	✓			
Bruggen-Rufi et al	✓		✓	✓		✓	✓	✓	✓	✓		
Gibson et al	✓	✓		✓			✓	✓				
Horton et al		✓				✓		✓	✓		✓	
Jarvis		✓						✓				
Learmonth	✓	✓	✓	✓				✓				
Kinnett-Hopkins et al		✓	✓	✓				✓				
Luker et al	✓	✓		✓		✓		✓	✓			
Masterson-Algar et al	✓	✓		✓	✓	✓	✓	✓	✓	✓	✓	
Nicholson		✓	✓	✓		✓		✓			✓	
Radford et al	✓		✓	✓	✓	✓	✓	✓	✓	✓		
Sadler et al	✓	✓						✓				
Simpson et al	✓	✓	✓				✓				✓	
Sturkenboom et al	✓	✓		✓	✓	✓	✓	✓	✓	✓		
Tielemans et al	✓	✓	✓	✓	✓			✓	✓			
Counts	30	29	17	17	6	10	29	35	10	6	5	3

Key: * = implied barriers as none explicitly stated

Some constructs had no determinants mapped:

- “Quality of delivery” (CFIF’s *moderating factors*) relates to the manner in which a teacher, volunteer, or staff member delivers a programme.
- “Design quality and packaging” relates to the perceived excellence in how the intervention is bundled, presented, and assembled. (CFIR; *intervention characteristics*).
- “Peer pressure” (CFIR; *outer setting)* relates to competitive pressure to deliver an intervention.
- “Planning”, “engaging”, “executing” (CFIR; *process*) relates to planning the implementation, how people were attracted to engaging with the process and how this was carried out.

### Stage two—understanding themes of barriers and facilitators in relation to implementation frameworks

Thematic analysis of extracted units of text revealed that barriers and facilitators were reported in similar ways across the 43 studies. This different perspective revealed six themes that demonstrated how researchers reported barriers and facilitators:

1. Non-adherence/adherence.
2. Perception of the intervention indicating a barrier/facilitator.
3. Attrition.
4. Trial-related barriers/facilitators.
5. Training barrier/facilitator.
6. Cost barrier/facilitator.

Most studies reported barriers (*n* = 30) and or facilitators (*n* = 29) related to adherence. Studies reported what study participants, carers and healthcare staff involved in delivering the intervention thought about the intervention, either negatively (*n* = 30) or positively (*n* = 35). Typically reported as “acceptability”, this theme was mapped to different constructs dependant on whether it was the patient’s perceptions or the clinicians. Patients’ reports of acceptability were mapped to “patient needs and resources” and clinicians’ perceptions mapped to “knowledge and beliefs of the intervention” in CFIR. Under half of studies (*n* = 17) described reasons for attrition and trial-related issues. Table 4 indicates which studies reported barriers and facilitators under which theme and the supplementary materials provide greater detail from each study alongside the themes together with a summary of the reported complex intervention.

## Discussion

The aim of this review was to identify the barriers and facilitators affecting the implementation of complex health interventions in adults with long-term neurological conditions (LTNC) in developed countries. This comprehensive and rigorous review resulted in the identification of 43 studies from eight countries, describing the problems and facilitators involved with the delivery of complex interventions.

Even though some researchers did not intend to focus on reporting barriers and facilitators, it was possible to identify these implementation issues as previously reported by Siemonsma [36]. Over 200 determinants (barriers and facilitators) were reported for interventions related to exercise, home-based rehabilitation, psychosocial and educational interventions, constraint-induced movement therapy, motor imagery, memory aids, self-management and continence training. Interventions were delivered to over 4000 people with LTNCs by over 400 rehabilitation health professionals: mostly occupational therapists and physiotherapists.

In order to be able to clearly describe implementation, barriers and facilitators were mapped onto constructs of two implementation research frameworks; CFIF [32] and CFIR [33]. Barriers and facilitators were mapped to most constructs, demonstrating they are wide ranging, which strengthens the usefulness of the frameworks, as others have found [34, 35, 90–92].

Six themes were identified that reflect how researchers currently tend to report barriers and facilitators. Most researchers reported barriers and facilitators in terms of “adherence” and “perceptions of the intervention”. Adherence can refer to the recipient and provider of an intervention and is regarded as an important determinant of intervention effectiveness [32]. Adherence (facilitator) and non-adherence (barrier) to intervention protocols were reported in 29 and 30 studies (respectively), indicating that researchers tend to routinely report these aspects. Most units of text within “adherence” were mapped to “facilitation strategies” in the CFIF, where positive strategies were undertaken to improve adherence to the intervention. Being able to identify these facilitators within existing studies will be helpful in the design of future similar studies.

The theme addressing “perceptions of the intervention” was reported positively and negatively in 35 and 30 studies (respectively) and primarily associated with the intervention’s acceptability. Acceptability is a broad term used to report a range of perceptions about an intervention from the perspective of recipients (people with LTNCs) and providers (clinicians) [93] but is also an important implementation issue [93, 94]. The concept of “acceptability” is important when considering responsiveness to an intervention [95] and especially so when moving from single studies to larger multi-centre trials where poor acceptability of interventions may affect their implementation [93]. Identifying issues related to intervention acceptability may help to reveal why some studies experienced implementation issues.

The reporting of both “perceptions of the intervention” and “adherence” is often required by publications. They indicate high-quality reporting and may explain the frequency of coverage in this review [96]. More recent guidance, for example; The TIDieR (Template for Intervention Description and Replication) Checklist [97], Standards for Quality Improvement Reporting Excellence (SQUIRE) [98] and Criteria for Reporting the Development and Evaluation of Complex Interventions in healthcare: revised guideline (CReDECI 2) [99] recommend this. These guidelines demonstrate the general agreement amongst many researchers that reporting the context of intervention delivery within research is important, but the increasing number of guidelines that now overlap is not necessarily more help [100].

As part of the process of developing new health interventions, researchers are encouraged to carry out process evaluations and feasibility studies that provide the opportunity to explore implementation issues comprehensively before progressing onto phase III trials [3, 101]. In the future, it is expected that more journals will encourage publication of process evaluations and implementation research, underpinned by a relevant theoretical framework. By doing so, researchers will need to become more aware of implementation research, resulting in increased reporting.

Overall, more barriers than facilitators were reported and links between determinants and recommendations for future implementation strategies are rarely made. This has been found elsewhere [18]. This may be because most studies did not explicitly set out to identify factors affecting the intervention implementation and may not have been aware of implementation research frameworks to support their identification or reporting. Identifying facilitators as well as barriers provides a useful starting point to develop and test implementation strategies. It is recommended that to understand the implementation of complex interventions for LTNCs, studies should identify barriers, facilitators, potential implementation strategies and methods to test them. This would promote understanding between determinants and the overall context [102].

The mapping process revealed 6 constructs without any barriers or facilitators mapped to them. These gaps relate to the quality of intervention delivery, design quality and packaging of the intervention, external (peer) pressure to implement the intervention and the process aspects of implementing the intervention (engaging, executing and reflecting). There may be several reasons for these gaps.

The interventions investigated in these studies may not have tapped these constructs, but it is unlikely that implementation of complex interventions for people with LTNCs went entirely smoothly, encountering no issues. The mapping procedure conducted in this review may have failed to assign some determinants to the appropriate constructs. However, it is more likely that researchers were unaware of the breadth of the context that “barriers and facilitators” emanate from or that these could inform future intervention implementation [30]. Historical publishing requirements, as mentioned above, may also have limited reporting. Whatever the reason, it is important to recognise these gaps have only been revealed by using implementation frameworks. In the future, researchers are recommended to use an implementation framework when examining implementation issues; if certain constructs in a framework do not attract determinants, this should be made explicit and explained. It is important for research teams to be aware of implementation research theory and to build this into study designs. Without measuring barriers and facilitators, it may be difficult to explain why an intervention works or not, and the chances of predicting success and designing strategies to ensure success are limited [30].

Whilst this review has shown it is possible to identify barriers and facilitators from studies that did not primarily intend to report them and then map them to two implementation research frameworks, studies using an implementation research framework were more logical and simplified the identification of barriers and facilitators. There is currently no standardised method for collecting or analysing data about implementation barriers and facilitators [30]. But the fact that it was possible to map multiple barriers and facilitators to so many constructs of CFIF and CFIR serves to validate their utility [103]. The two frameworks used in this review offer little guidance on how to identify barriers and facilitators. The lack of differentiation between implementation theories, models and frameworks [30, 33, 104] adds to the difficulty in choosing one to use dependent on the study [30, 105, 106]. But being uninformed does not seem a suitable reason for risking repeating known implementation mistakes [30, 106, 107]. CFIR has online resources sharing knowledge aimed at developing tools [108]. To progress this work further, researchers should engage in moving theories forward to include practical guidance and tools [30, 94, 107].

Trial-related issues are described less often in the older papers in this review. The Medical Research Council (MRC) guidance on process evaluations of complex interventions reinforces that rehabilitation researchers should examine and report barriers and facilitators throughout intervention development [109] and not leave this until translation into practice. There is acknowledgement of differences between implementing rehabilitation into clinical practice compared with a trial, but little is known about the research setting [16, 84]. Even though the MRC guidance on developing complex interventions and process evaluations has been widely cited over the past 9 years, the rehabilitation research community has been slow to respond. The more recent papers included in this review who have examined the research context in more detail may reflect increased awareness of the value of describing what affects intervention delivery and other trial aspects such as preparing a site ready for recruitment, training therapists to deliver an intervention and understanding the perceptions of a wider range of stakeholders, not only patients receiving the intervention.

By addressing the implementation of rehabilitation early in an intervention’s development, trialists can provide assurance about intervention effectiveness by demonstrating that it has been implemented as planned, thus preventing type III errors [29]. The return on investment into rehabilitation research and outcomes for people with LTNCs may improve by understanding the implementation of interventions early on. Eventually*,* this may help policy makers and commissioners understand how an intervention will work in a range of contexts with different populations and improve their confidence to warrant funding new interventions [27, 28].

One main strength of this review is the thorough and systematic search methodology. Using broad inclusion criteria to maximise the identification of published research strengthened the process. The review has focussed on a range of important implementation issues that have not been included in previous literature reviews for LTNCs. However, more stringent inclusion and exclusion criteria could have distinguished more formal research from other studies based in clinical settings.

Theoretical frameworks from implementation research underpinned the review and facilitated in-depth, two-stage data analysis. Using the CFIF and CFIR frameworks together allowed an understanding of how they relate to each other. Parallels and variances in language used were revealed, which inform theoretical development [33, 110]. The use of two complementary theoretical frameworks, each with a different focus on implementation, provided structure to the analysis [105, 111] and helped to ensure the results are relevant across settings [111].

The team approach to this review helped to address bias through discussions and reaching consensus. The author’s background as a practising clinician was made explicit as a potential source of bias during thematic analysis and data interpretation [112].

Identifying relevant text within studies was a lengthy process because the language and definitions used were not standardised, requires subjective judgement and is open to interpretation. Other researchers could have reached a different conclusion and mapped determinants to different constructs. The findings that some constructs had no barriers or facilitators mapped to them could be because of the mapping process itself. The lack of budget for foreign language translations meant the review was limited to the English language.

## Conclusions

This review has described the barriers and facilitators identified in studies implementing complex interventions for people with LTNCs. Studies adopting an implementation framework simplified the identification of barriers and facilitators, an important consideration for busy researchers. In the development of a new complex intervention, it is vital to learn from previous experiences to prevent unnecessary repetitions of implementation failure at both trial and service provision levels. Therefore, researchers and service providers should be cognizant of and utilise implementation theory and implementation frameworks to guide the identification and reporting of implementation issues in future studies. Clinicians should look to studies that have utilised implementation theory and make use of reported strategies for overcoming implementation issues.

The information gleaned in this review was used in an implementation strategy, where occupational therapists were trained to deliver a new early specialist vocational rehabilitation intervention for TBI in the NHS in England, in a feasibility randomised controlled trial [24, 113]. The barriers and facilitators identified in this review helped to inform trainers and mentors in supporting the therapists [114]. Other researchers may also find the information useful to inform study design and understand, for instance, what support might be required by clinicians delivering an intervention.

## Recommendations for researchers

- Investigate barriers and facilitators early in the development of rehabilitation interventions.
- Use an implementation framework to guide the investigation.
- Explore the findings of similar research to avoid unnecessary repetition of implementation issues.
- Describe barriers and facilitators in sufficient detail for others to make useful comparisons.

## Supplementary Information

**Additional file 1.**

## Data Availability

All data generated or analysed during this study are included in this published article [and its supplementary information files.

## Abbreviations

LTNC: Long-term neurological condition
CFIF: Comprehensive framework for implementation fidelity
CFIR: Consolidated framework for implementation research
TBI: Traumatic brain injury
VR: Vocational rehabilitation
MRC: Medical Research Council
