# Supplementary material for: Factors affecting the delivery of complex rehabilitation interventions in research with neurologically impaired adults: a systematic review
Source: Syst Rev. 2020 Nov 25;9:268. doi: 10.1186/s13643-020-01508-1 (PMC7690089; doi:10.1186/s13643-020-01508-1)
Supplement: Supplementary file 1 — Additional file 1. [file 13643_2020_1508_MOESM1_ESM.docx]

**MEDLINE Search Terms**

1. (Barrier* or Block* or Hurdle* or barricad* or Obstruct* or obstacle* or Facilitat* or enable* or Support* or Encourag* or challeng* or problem* or threat*).mp. [mp=title, abstract, original title, name of substance word, subject heading word, floating sub-heading word, keyword heading word, protocol supplementary concept word, rare disease supplementary concept word, unique identifier, synonyms]

2. (Implement* or Applicat* or adopt* or uptake or Apply or Execut* or Instigat* or Carry out or Employ* or Perform* or Usage or Practice or Enactment or Fulfil*).mp. [mp=title, abstract, original title, name of substance word, subject heading word, floating sub-heading word, keyword heading word, protocol supplementary concept word, rare disease supplementary concept word, unique identifier, synonyms]

3. Migraine Disorders/px, rh [Psychology, Rehabilitation]

4. Huntington Disease/ or Neurodegenerative Diseases/

5. Postpoliomyelitis Syndrome/px, rh [Psychology, Rehabilitation]

6. Muscular Atrophy/ or Charcot-Marie-Tooth Disease/ or Friedreich Ataxia/

7. Myasthenia Gravis/px, rh [Psychology, Rehabilitation]

8. Neuromuscular Diseases/px, rh [Psychology, Rehabilitation]

9. Brain Injuries/px, rh [Psychology, Rehabilitation]

10. Dementia/

11. Cerebral Palsy/px, rh [Psychology, Rehabilitation]

12. Amyotrophic Lateral Sclerosis/ or Motor Neuron Disease/ or "motor adj neuron* disease".mp. [mp=title, abstract, original title, name of substance word, subject heading word, floating sub-heading word, keyword heading word, protocol supplementary concept word, rare disease supplementary concept word, unique identifier, synonyms]

13. Stroke/px, rh [Psychology, Rehabilitation]

14. Muscular Dystrophies/px, rh [Psychology, Rehabilitation]

15. Multiple Sclerosis/ or Nervous System Diseases/ or Spinal Cord Neoplasms/

16. Spinal Cord Injuries/px, rh [Psychology, Rehabilitation]

17. Parkinson Disease/px, rh [Psychology, Rehabilitation]

18. 3 or 4 or 5 or 6 or 7 or 8 or 9 or 10 or 11 or 12 or 13 or 14 or 15 or 16 or 17

19. Rehabilitation/ or occupational therapy/ or allied health personnel/ or physical therapy modalities/ or rehabilitation nursing/ or (speech and language).mp. or behavioral medicine/ or "Physical and Rehabilitation Medicine"/ or therapist.mp. or complex intervention.mp. [mp=title, abstract, original title, name of substance word, subject heading word, floating sub-heading word, keyword heading word, protocol supplementary concept word, rare disease supplementary concept word, unique identifier, synonyms]

20. Parents/

21. Child/rh, th [Rehabilitation, Therapy]

22. Schools/

23. Medical Records Systems, Computerized/ or Electronic Health Records/ or Medical Records/

24. Guideline Adherence/ or Guideline/ or Practice Guideline/

25. Africa/

26. Malaysia/

27. Indonesia/

28. India/

29. South America/

30. child*.mp. [mp=title, abstract, original title, name of substance word, subject heading word, floating sub-heading word, keyword heading word, protocol supplementary concept word, rare disease supplementary concept word, unique identifier, synonyms]

31. (school* or preschool*).mp. [mp=title, abstract, original title, name of substance word, subject heading word, floating sub-heading word, keyword heading word, protocol supplementary concept word, rare disease supplementary concept word, unique identifier, synonyms]

32. (Barrier* or Block* or Hurdle* or barricad* or Obstruct* or obstacle* or Facilitat* or enable* or Support* or Encourag* or challeng* or problem* or threat*).mp. adj5 (Rehabilitation/ or occupational therapy/ or allied health personnel/ or physical therapy modalities/ or nursing/ or rehabilitation nursing/ or (speech and language).mp. or behavioral medicine/ or "Physical and Rehabilitation Medicine"/ or therapist.mp. or complex intervention.mp.)

33. (Implement* or Applicat* or adopt* or uptake or Apply or Execut* or Instigat* or Carry out or Employ* or Perform* or Usage or Practice or Enactment or Fulfil*).mp. adj5 (Rehabilitation/ or occupational therapy/ or allied health personnel/ or physical therapy modalities/ or nursing/ or rehabilitation nursing/ or (speech and language).mp. or behavioral medicine/ or "Physical and Rehabilitation Medicine"/ or therapist.mp. or complex intervention.mp.)

34. ((Barrier* or Block* or Hurdle* or barricad* or Obstruct* or obstacle* or Facilitat* or enable* or Support* or Encourag* or challeng* or problem* or threat*) adj5 (Implement* or Applicat* or adopt* or uptake or Apply or Execut* or Instigat* or Carry out or Employ* or Perform* or Usage or Practice or Enactment or Fulfil*)).mp.

35. ((Barrier* or Block* or Hurdle* or barricad* or Obstruct* or obstacle* or Facilitat* or enable* or Support* or Encourag* or challeng* or problem* or threat*) adj4 (Implement* or Applicat* or adopt* or uptake or Apply or Execut* or Instigat* or Carry out or Employ* or Perform* or Usage or Practice or Enactment or Fulfil*)).mp.

36. ((Barrier* or Block* or Hurdle* or barricad* or Obstruct* or obstacle* or Facilitat* or enable* or Support* or Encourag* or challeng* or problem* or threat*) adj3 (Implement* or Applicat* or adopt* or uptake or Apply or Execut* or Instigat* or Carry out or Employ* or Perform* or Usage or Practice or Enactment or Fulfil*)).mp.

37. ((Barrier* or Block* or Hurdle* or barricad* or Obstruct* or obstacle* or Facilitat* or enable* or Support* or Encourag* or challeng* or problem* or threat*) adj2 (Implement* or Applicat* or adopt* or uptake or Apply or Execut* or Instigat* or Carry out or Employ* or Perform* or Usage or Practice or Enactment or Fulfil*)).mp.

38. 37 and 18 and 19

39. 36 and 18 and 19

40. 35 and 18 and 19

41. 34 and 18 and 19

42. 1 and 2 and 18 and 19

43. 42 not 20 not 21 not 22 not 23 not 30 not 31

44. Feasibility Studies/ or feasib*.mp.

45. (robotic* or "virtual reality").mp. [mp=title, abstract, original title, name of substance word, subject heading word, floating sub-heading word, keyword heading word, protocol supplementary concept word, rare disease supplementary concept word, unique identifier, synonyms]

46. (43 and 44) not 45

47. limit 46 to (humans and "all adult (19 plus years)")

48. 43 not 45

49. limit 48 to (humans and "all adult (19 plus years)")

50. (robotic* or "virtual reality" or virtual*).mp. [mp=title, abstract, original title, name of substance word, subject heading word, floating sub-heading word, keyword heading word, protocol supplementary concept word, rare disease supplementary concept word, unique identifier, synonyms]

51. 43 not 50

52. limit 51 to (humans and "all adult (19 plus years)")

53. (tele* or telerehabilitation or telehealth).mp. [mp=title, abstract, original title, name of substance word, subject heading word, floating sub-heading word, keyword heading word, protocol supplementary concept word, rare disease supplementary concept word, unique identifier, synonyms]

54. 51 not 53

55. limit 54 to (humans and "all adult (19 plus years)")
